# Charge Scheduling of an Energy Storage System under Time-of-Use Pricing and a Demand Charge

**DOI:** 10.1155/2014/937329

**Published:** 2014-08-13

**Authors:** Yourim Yoon, Yong-Hyuk Kim

**Affiliations:** ^1^Department of Computer Engineering, College of Information Technology, Gachon University, 1342 Seongnamdaero, Sujeong-gu, Seongnam-si, Gyeonggi-do 461-701, Republic of Korea; ^2^Department of Computer Science & Engineering, Kwangwoon University, 20 Kwangwoon-ro, Nowon-gu, Seoul 139-701, Republic of Korea

## Abstract

A real-coded genetic algorithm is used to schedule the charging of an energy storage system (ESS), operated in tandem with renewable power by an electricity consumer who is subject to time-of-use pricing and a demand charge. Simulations based on load and generation profiles of typical residential customers show that an ESS scheduled by our algorithm can reduce electricity costs by approximately 17%, compared to a system without an ESS and by 8% compared to a scheduling algorithm based on net power.

## 1. Introduction

An energy storage system (ESS) is a system that is capable of absorbing energy, storing it for a period of time, and then returning it for use. In an electrical grid, an ESS can be used to match supply and demand. The ESS is charged when demand is low and discharged when demand is high. Thus, the overall energy efficiency of a system is improved, and the energy flow from the electrical grid connected to the system is stabilized. Reliability is a key issue in the effective use of renewable energy and in smart grids, and thus the demand for ESSs is increasing [1].

An ESS acts as a buffer between a generator and its load. Renewable energy sources often generate power during off-peak periods or when demand for energy is low. ESSs enable better integration of renewable energy sources into an electrical grid by (time-shifting) the generated power and smoothing out spikes in demand. Power producers can benefit from a more predictable generation requirement, which can improve revenue. Installing an ESS can enable industrial, commercial, or residential end-users to improve the quality and reliability of their power supply and to reduce their electricity costs and can act as a back-up power source [2, 3].

Dynamic pricing of electricity is being facilitated by new technologies such as smart meters. A form of dynamic pricing that is being adopted in many areas is known as time-of-use (TOU) pricing, in which electricity prices are set for a fixed period. Energy providers use TOU pricing to drive down demand at peak periods by using high prices to influence customers' consumption rather than more invasive controls such as dynamic or passive demand response mechanisms, or even power cuts [4, 5]. Typically TOU prices do not change more than twice a year, but a TOU tariff is likely to have two or three price levels (e.g., “off-peak”, “mid-peak”, and “on-peak”) where the price is determined by the time of day. Customers can be expected to vary their usage in response to this price information and manage their energy costs by shifting their usage to a lower cost period. ESSs will play an important role in residential areas with a dynamic pricing policy. By storing energy during low off-peak price periods and using the stored energy when the price is high, consumers can avoid paying high rates.

In addition to charges based on usage, an electricity bill may include a* demand charge*, which is determined by the maximum energy capacity available to a customer, whether or not it is actually used. The demand charge is billed as a fixed rate that is calculated on a per kW basis. This charge is based on the premise that commercial customers and other large users should pay a share of the infrastructure costs associated with the maintenance of capacity [6]. We will consider both TOU pricing and demand charges [7].

Many problems related to the scheduling of the charging and discharging of an ESS have been studied recently [8–12]. Various optimization techniques can be applied to the operation of ESSs. The most frequently used method is dynamic programming, which was used by Maly and Kwan [13]. They tried to minimize electricity cost for an ESS with a given battery capacity, without unnecessarily reducing battery life. Van de Ven et al. [14] aimed to minimize the capital cost of an ESS subject to user demand and prices, as a Markov decision process, which can be solved using dynamic programming. Koutsopoulos et al. [15] addressed the optimal ESS control problem from the point of view of a utility operator and solved the off-line problem over a finite period by dynamic programming. Romaus et al. [16] investigated stochastic dynamic programming for energy management of a hybrid ESS for electric vehicles. They aimed to control the power flow to the ESS online, while taking into account the stochastic influences of traffic and the driver. Huang and Liu [17] applied adaptive dynamic programming to the management of a residential ESS, with an emphasis on domestic electricity storage systems. Their scheme was designed to learn during operation as the environment of the ESS changes unpredictably.

There have also been a number of studies using other scheduling methods. Youn and Cho [18] used linear programming to pursue optimal operation of an energy storage unit installed in a small power station. Hu et al. [19] used sequential quadratic programming to operate on ESS under real-time changes to the electricity price, so as to maximize profits. Nonlinear programming techniques were adopted by Rupanagunta et al. [20] to design an optimal controller for charge and discharge processes in ESSs, with the objective of minimizing the operating costs of the storage facility. Yoo et al. [21] used a Kalman filter to increase predictability in controlling the power flows between the components of an energy management system for a grid-connected residential photovoltaic (PV) system combined with an ESS under critical peak pricing. Lee [22] used multipass iteration particle swarm optimization to determine the operating schedule of an ESS for an industrial TOU-rate user who is also operating wind turbine generators. Gallo et al. [23] used a hybrid optimization technique to determine values of the battery parameters required for an ESS operated by a smart grid management system. Their method combines stochastic and deterministic elements within a computationally efficient algorithm.

In this paper we describe a real-coded genetic algorithm (RCGA) for scheduling ESS charging and discharging. Genetic algorithms (GAs) were used by Monteiro et al. [24] for short-term forecasting of the energy output of a PV plant. They applied data mining techniques to historical forecasts of weather variables. The GA was used to make spot forecasts of power output from PV plants. We use an RCGA to schedule ESS operations under TOU pricing with a demand charge, when a supply of renewable energy, wind or solar energy, is available.

The remainder of the paper is organized as follows. In Section 2 we describe the ESS scheduling problem under TOU pricing with a demand charge, when renewable electricity is available. In Section 3 we describe an RCGA that addresses this problem. In Section 4 we present the simulation results, and draw conclusions in Section 5.

## 2. ESS Scheduling Problem under TOU Pricing with a Demand Charge

The formulation of our problem is similar to that of Lee [22], but we aim to optimize a daily, rather than a monthly, bill. Other studies have dealt with optimization problems under TOU pricing. Cao et al. [25] proposed an intelligent method to control EV charging loads in response to TOU price in a regulated market. Lee and Chen [26] formulated the problem of determining the optimal contract capacities and optimal sizes of ESSs for customers using a TOU rate.

Notations/expressions in the appendix summarize the notation and some of the expressions used in this study. The load *l*_*i*_ is the amount of energy used during time interval *i*, and *g*_*i*_ is the amount of energy generated over the same period. The residual energy in the battery at the end of interval *i* is *x*_*i*_. We set the length of a time interval to one hour. The energy supplied to the battery during time interval *i* is *x*_*i*_ − *x*_*i*−1_ and the net energy drawn from the grid is *x*_*i*_ − *x*_*i*−1_ + *l*_*i*_ − *g*_*i*_. Thus, the cost of energy over the time interval *i* is (*x*_*i*_ − *x*_*i*−1_ + *l*_*i*_ − *g*_*i*_)*p*_*i*_, where *p*_*i*_ is the price set for that interval. The possibility of compensation tariff for feed-in electricity is not considered in this study. If such a tariff is high, scheduling will favor feeding electricity into the grid. However, the trend in smart grid pricing is to encourage residential users to conserve any electricity that they generate, so feed-in tariffs are likely to become very low or zero, which is what we assume. In this case, the total cost of energy over *T* time intervals is ∑_*i*=1_^*T*^ *I*(*x*_*i*_ − *x*_*i*−1_ + *l*_*i*_ − *g*_*i*_ > 0) (*x*_*i*_ − *x*_*i*−1_ + *l*_*i*_ − *g*_*i*_)*p*_*i*_, where *I* is the indicator function. We use twenty-four hour data and set *T* to 24.

The total cost of electricity is the sum of the energy charge and the demand charge, which is the product of the fixed rate p* and the peak demand, and can thus be written max⁡_1≤*i*≤*T*_{*x*_*i*_ − *x*_*i*−1_ + *l*_*i*_ − *g*_*i*_}p*. The problem of minimizing the total cost of electricity can now be expressed as follows:

(1) Minimize  ∑i=1TI(xi−xi−1+li−gi>0)(xi−xi−1+li−gi)pi    +max⁡1≤i≤T{xi−xi−1+li−gi}p∗,subject  to    0≤xi≤C, i=1,2,…,T,    −Pd≤xi−xi−1≤Pc, i=1,2,…,T,

where *C* is the total battery capacity, *P*_*d*_ is the battery discharge power, and *P*_*c*_ is the battery charge power. The value of *x*_*i*_ cannot exceed the battery capacity, and the net amount of energy *x*_*i*_ − *x*_*i*−1_ flowing in or out of the battery should lie in the range [−*P*_*d*_, *P*_*c*_].

## 3. Real-Coded Genetic Algorithm

GAs that are based on real number representation are called real-coded GAs (RCGAs) [27]. Real coding was first used in specific applications, such as chemometric problems and in using metaoperators to find the most appropriate parameters for a standard GA [27]. Subsequently, RCGAs have mainly been used in numerical optimization problems over continuous domains [28–31].

In our RCGA, a population consisting of *N*/2 pairs are randomly selected from a population of *N*, and crossover and mutation operators are applied to each pair to generate *N*/2 offspring. Both parents and offspring are ranked and the best *N* become the next generation. We use a population of 100, and our RCGA terminates after 2,000 generations.

### 3.1. Encoding

Our RCGA is encoded using an array of *T* real numbers. Our approach differs from a typical real encoding in that each gene *x*_*i*_ has its own range of real values that are determined by the value of its left-sided gene *x*_*i*−1_. The following two constraints must be satisfied by each value of *x*_*i*_:

(2) $$\begin{array}{c} 0\leq x_{i}\leq C,\\ -P_{d}\leq x_{i}-x_{i-1}\leq P_{c}⟺x_{i-1}-P_{d}\leq x_{i}\leq x_{i-1}+P_{c}. \end{array}$$

Therefore *x*_*i*_ must satisfy the following expression in *x*_*i*−1_:

(3) $$\begin{array}{c} \max\left(0,x_{i-1}-P_{d}\right)\leq x_{i}\leq \min\left(C,x_{i-1}+P_{c}\right). \end{array}$$

### 3.2. Evaluation

The objective function for the problem is used as the evaluation function of the RCGA: ∑_*i*=1_^*T*^ *I*(*y*_*i*_ − *y*_*i*−1_ + *l*_*i*_ − *g*_*i*_ > 0) (*y*_*i*_ − *y*_*i*−1_ + *l*_*i*_ − *g*_*i*_)*p*_*i*_ + max⁡_1≤*i*≤*T*_{*y*_*i*_ − *y*_*i*−1_ + *l*_*i*_ − *g*_*i*_}p*. Because this is a minimization problem, solutions with smaller objective values are more likely to survive.

### 3.3. Initialization

Initially 100 solutions are generated, satisfying the feasibility constraint of (3). For each *i* (*i* = 1,2,…, *T*), a real number is randomly chosen over the interval [max⁡(0, *x*_*i*−1_ − *P*_*d*_), min⁡(*C*, *x*_*i*−1_ + *P*_*c*_)]. Each solution generated by this procedure corresponds to an available ESS schedule.

### 3.4. Crossover Operator

We use the crossover operator BLX-*α* [32, 33], where *α* is a nonnegative real-valued parameter. This operator produces *z*_*k*_ = (*z*_1_, *z*_2_,…, *z*_*n*_) offspring, where *z*_*i*_ is a random number chosen over the interval [*C*_min⁡_ − *αI*, *C*_max⁡_ + *αI*], where *C*_max⁡_ = max⁡(*x*_*i*_, *y*_*i*_), *C*_min⁡_ = min⁡(*x*_*i*_, *y*_*i*_), and *I* = *C*_max⁡_ − *C*_min⁡_. The value of *α* is set to 0.5 in our RCGA. To ensure that each gene satisfies (3), BLX-*α* is modified so that it accepts random real numbers over the interval [max⁡(0, *x*_*i*−1_ − *P*_*d*_, *C*_min⁡_ − *αI*), min⁡(*C*, *x*_*i*−1_ + *P*_*c*_), *C*_max⁡_ + *αI*)], instead of the interval [*C*_min⁡_ − *αI*, *C*_max⁡_ + *αI*].

### 3.5. Mutation Operator

In Gaussian mutation [34], the *i*th parameter *x*_*i*_ of an individual is mutated by *x*_*i*_ = *x*_*i*_ + *N*(0, *σ*_*i*_) at a mutation rate *p*_*m*_, where *N*(0, *σ*_*i*_) is an independent random Gaussian number with a mean of zero and a standard deviation of *σ*_*i*_. In our RCGA, *σ*_*i*_ is set to min⁡(*C*, *x*_*i*−1_ + *P*_*c*_) − max⁡(0, *x*_*i*−1_ − *P*_*d*_), which is the magnitude of the range of feasible solutions. If a mutated value is not in [min⁡(*C*, *x*_*i*−1_ + *P*_*c*_), max⁡(0, *x*_*i*−1_ − *P*_*d*_)], then it is replaced by min⁡(*C*, *x*_*i*−1_ + *P*_*c*_) or max⁡(0, *x*_*i*−1_ − *P*_*d*_), whichever is the closest, to produce a feasible solution. If *i* < *j* ≤ *T*, then changes to *x*_*i*_ can affect the feasibility of *x*_*j*_. Thus, values of *x*_*j*_ which are not in [min⁡(*C*, *x*_*j*−1_ + *P*_*c*_), max⁡(0, *x*_*j*−1_ − *P*_*d*_)] are similarly replaced by the closer of min⁡(*C*, *x*_*j*−1_ + *P*_*c*_) or max⁡(0, *x*_*j*−1_ − *P*_*d*_). The value of *p*_*m*_ is set to 0.1/*T*.

## 4. Simulation Results

### 4.1. Problems Instances

The load profile that we will use is a residential customer profile provided by NorthWestern Energy [35] and is based on data for 1992 and 1993. The company's Load Vision profiling software was used to construct profiles for typical diversified residential loads on weekdays and at weekends for each season and three weather scenarios. The portion of the data that we used in this study is presented as Table 1.

Hourly solar generation data were obtained using PVWatts, developed by the National Renewable Energy Laboratory (NREL). This calculator predicts the energy production of residential and small commercial PV installations, based on hourly data for sunny and cloudy days in Helena, a city in the northwestern United States. The specifications of the PV system that we consider are listed in Table 2.

Typical TOU prices were generated by simulations using three price levels, for summer and winter, based on the TOU pricing models of several utility companies. We consider two daily rates of demand charge, 20 cents/kW (low) and 30 cents/kW (high). The TOU pricing model that we have constructed is given in Table 3.

We consider a battery with a total capacity of 2 kWh, but only 1.8 kWh is used to extend battery life. The maximum rate of charge and discharge is around to be 0.6 kW. Thus, we set *C* to 1.8, and values of *P*_*c*_ and *P*_*d*_ of 0.6 kW are used in the problem formulation.

### 4.2. Results and Discussion

We compared our RCGA with a net-power-based algorithm (NPB) which charges or discharges the battery to make up the difference between the power generated and the load. This naive algorithm does not consider the electricity price at all.

The simulation results are shown in Table 4. All the algorithms run in under a second on an Intel Xeon CPU E5530 @ 2.40 GHz. A run of RCGA took 0.18 seconds.

The results in Table 4 show that our RCGA always outperforms the NPB. The maximum benefit is 11% in Cases 7, 8, and 16, and the minimum is 4% in Case 2. The RCGA performed better in the winter than in the summer. This result can be explained by the difference in the summer and winter price schedules. In summer, the peak period usually occurs at a time when PV energy is plentiful, and so the power drawn from the grid is easily reduced, without the need for an elaborate algorithm. In winter, there is much less PV energy available during peak periods, making the RCGA more effective. We also show the effectiveness of the RCGA by comparing it with another optimization method in the appendix.

**Figure 1:**
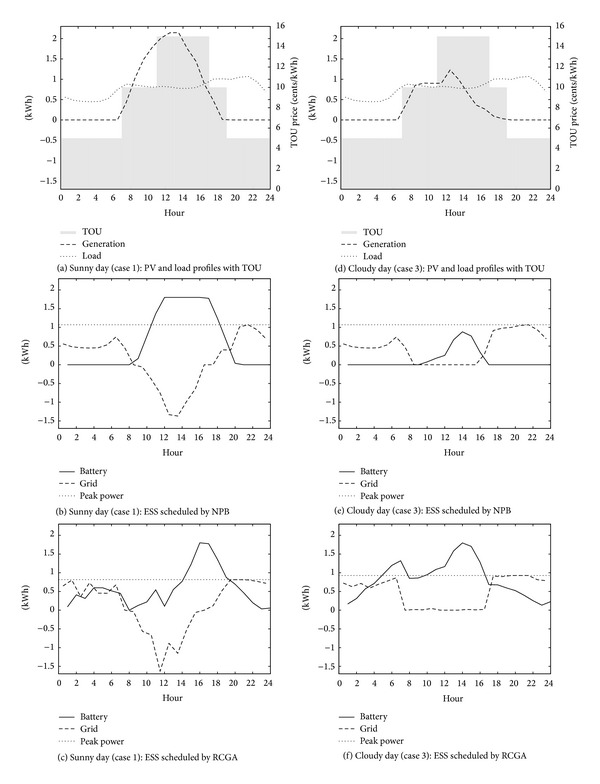
Simulated battery schedules for summer weekdays.

**Figure 2:**
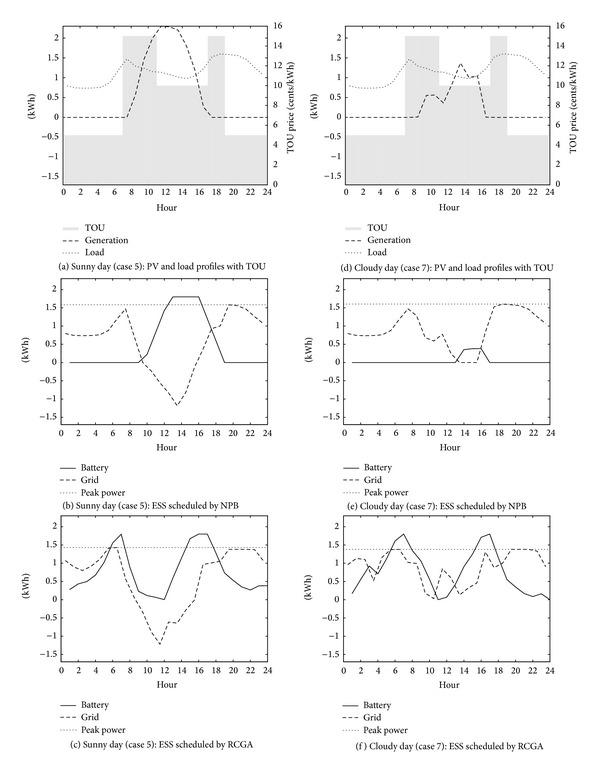
Simulated battery schedules for winter weekdays.

Figures 1 and 2 show simulated levels of battery charge which are produced by the NPB and RCGA in the winter and the summer scenarios. Typical PV and load profiles with TOU prices for each season and weather scenario are shown in Figures 1(a), 1(d), 2(a), and 2(d). The other figures show battery charge, the average and peak power drawn from the grid. The NPB charges the battery when the generation exceeds the load and discharges otherwise. The ESS operated on the NPB schedule charges the battery in the daytime when the sun blazes and discharges the battery in the evening when the sun sets, regardless of the season and the weather. However, the RCGA optimizes the ESS charge schedule to minimize the electricity cost under various constraints and a given pricing policy. Thus, the ESS operated on the RCGA schedule charges and discharges the battery dynamically depending on the season and the weather. The figures show that the RCGA schedules reduce both the peak power and the purchase of electricity (i.e., grid power) during on-peak periods.

## 5. Conclusion

We have developed an RCGA for ESS charge scheduling, which is especially important for electricity customers who have to contend with dynamic pricing. We considered TOU pricing with a demand charge when electricity is supplied to a customer with their own renewable energy generation facility. The scheduling problem for this scenario was formally defined, and the RCGA was used to develop a novel approach to charge scheduling. Experiments using the load and generation profiles of typical residential customers showed that scheduling by the RCGA reduced both the peak power consumption and the purchase of electricity during on-peak periods. This suggests that charge scheduling using an RCGA can help to reduce customers' electricity bills.

Neither battery efficiency nor the capital cost of a storage system was considered in this study, although these factors can clearly affect overall cost. Further studies that consider these factors are needed. It would also be interesting to investigate how our RCGA performs under more dynamic pricing schemes such as real-time pricing, which are a part of many smart grid scenarios.

## Figures and Tables

**Table 1:** Residential customer load profile data used in this study.

Seasons	Summer (Jun.–Sep.)
	Winter (Dec.–Feb.)

Types of day	Weekday
	Weekend

Weather scenarios	Normal

**Table 2:** PV system specifications.

DC rating	3 kW
DC to AC derating factor	0.77
Array type	Fixed tilt
Array tilt	46.6° (latitude)
Array azimuth	180.0° (true south)

**Table 3:** Time-of-use prices used in this study (USD).

Hour (from–to)	Summer (cents/kWh)	Winter (cents/kWh)
0-1	5	5
1-2	5	5
2-3	5	5
3-4	5	5
4-5	5	5
5-6	5	5
6-7	5	5
7-8	10	15
8-9	10	15
9-10	10	15
10-11	10	15
11-12	15	10
12-13	15	10
13-14	15	10
14-15	15	10
15-16	15	10
16-17	15	10
17-18	10	15
18-19	10	15
19-20	5	5
20-21	5	5
21-22	5	5
22-23	5	5
23-24	5	5

p*	Demand charge rate: 20 (low), 30 (high) (cents/kW)	

**Table 4:** Comparison of simulation results for a single day.

Instance					NO-ESS	NPB		RCGA	
Case	Rate	Season	Weather	Day type	Cost	Cost	Saving	Ave. cost (Std.)	Saving
1	Low	Summer	Sunny	Weekday	83.69	68.76	18	63.87 (0.63)	24
2	Low	Summer	Sunny	Weekend	82.28	65.42	20	62.90 (0.61)	24
3	Low	Summer	Cloudy	Weekday	104.64	91.42	13	86.19 (1.09)	18
4	Low	Summer	Cloudy	Weekend	111.40	107.71	3	98.32 (1.09)	12
5	Low	Winter	Sunny	Weekday	185.43	161.05	13	150.08 (0.97)	19
6	Low	Winter	Sunny	Weekend	176.93	152.00	14	140.67 (1.22)	20
7	Low	Winter	Cloudy	Weekday	225.68	221.86	2	197.37 (0.74)	13
8	Low	Winter	Cloudy	Weekend	233.26	233.26	0	207.44 (0.69)	11
9	High	Summer	Sunny	Weekday	94.37	79.44	16	71.75 (0.72)	24
10	High	Summer	Sunny	Weekend	92.47	75.44	18	70.43 (0.76)	24
11	High	Summer	Cloudy	Weekday	115.32	102.10	11	96.32 (1.20)	16
12	High	Summer	Cloudy	Weekend	121.42	117.73	3	108.42 (1.31)	11
13	High	Winter	Sunny	Weekday	201.45	176.88	12	163.75 (1.26)	19
14	High	Winter	Sunny	Weekend	192.56	166.84	13	153.75 (1.33)	20
15	High	Winter	Cloudy	Weekday	241.70	237.88	2	211.49 (1.09)	12
16	High	Winter	Cloudy	Weekend	248.89	248.89	0	221.26 (0.90)	11

NO-ESS is the cost with no ESS.

The NPB algorithm charges the battery when the generated power exceeds the load and discharges otherwise.

RCGA is our real-coded genetic algorithm (the average costs are obtained over 100 runs).

All costs are in US cents, and savings are percentages.

The saving for Algorithm *A* is obtained using the formula, 100 × (Cost_NO-ESS_ − Cost_*A*_)/Cost_NO-ESS_, where Cost_*A*_ is the electricity cost incurred by Algorithm *A*.

**Table 5:** Comparison between MSM and RCGA.

Case	MSM		RCGA	
	Ave. cost (Std.)	Saving	Ave. cost (Std.)	Saving
1	67.31 (0.60)	20	63.87 (0.63)	24
2	65.99 (0.60)	20	62.90 (0.61)	24
3	91.59 (0.77)	12	86.19 (1.09)	18
4	100.04 (0.71)	10	98.32 (1.09)	12
5	155.49 (0.84)	16	150.08 (0.97)	19
6	146.52 (0.96)	17	140.67 (1.22)	20
7	202.14 (0.77)	10	197.37 (0.74)	13
8	212.02 (0.74)	9	207.44 (0.69)	11
9	76.37 (0.82)	19	71.75 (0.72)	24
10	75.00 (0.73)	19	70.43 (0.76)	24
11	102.51 (0.78)	11	96.32 (1.20)	16
12	110.77 (0.87)	9	108.42 (1.31)	11
13	170.30 (1.06)	15	163.75 (1.26)	19
14	160.89 (1.04)	16	153.75 (1.33)	20
15	217.18 (0.83)	10	211.49 (1.09)	12
16	226.66 (0.88)	9	221.26 (0.90)	11

The average costs in US cents are obtained over 100 runs.

## Appendix

### Effectiveness of the Proposed RCGA

To check the performance of our RCGA, we ran a multistart method [36] which starts with many different initial solutions and returns the best among them. We denote by MSM the algorithm that randomly generates 10^7^ solutions as given in Section 3.3 and produces the best as its final solution.  Table 5 compares the performance of the MSM and the RCGA. A run of the MSM took 5.4 seconds, which is 30 times longer than that of the RCGA. The MSM showed better performance than the NPB overall, but it was inferior to the RCGA for all the cases. The results show the effectiveness of our genetic search process.

## Notations/Expressions

*l*_*i*_: Load during time interval *i*
*g*_*i*_: Energy generated during time interval *i*
*x*_*i*_: Residual energy in the battery at the end of time interval *i*
*x*_*i*_ − *x*_*i*−1_: Energy supplied to the battery during time interval *i*
*x*_*i*_ − *x*_*i*−1_ + *l*_*i*_ − *g*_*i*_: Net energy drawn from the grid during time interval *i*
*p*_*i*_: Energy price set for time interval *i*
*p**: Fixed price
(*x*_*i*_ − *x*_*i*−1_ + *l*_*i*_ − *g*_*i*_)*p*_*i*_: Cost of energy over time interval *i*
*T*: Number of time intervals = 24
*C*: Battery capacity
*P*_*c*_: Battery charge power
*P*_*d*_: Battery discharge power
*I*(·): Indicator function such that *I*(true) = 1 and *I*(false) = 0.
